# Surviving the First Shift as a Resident Doctor in Psychiatry: A Quality Improvement Project Analysing Feedback for the New and Improved Resident Doctors Handbook in Psychiatry in North Wales

**DOI:** 10.1192/bjo.2026.11468

**Published:** 2026-06-30

**Authors:** Aneeq Rao, Wamiqur Rehman Gajdhar, Ashna Anil, Pramestha Khoju Shrestha, Asha Dhandapani

**Affiliations:** 1 Betsi Cadwaladr University Health Board, Wrexham, United Kingdom; 2 Cardiff and Vale HealthCare, Cardiff, United Kingdom; 3 Betsi Cadwaladr University Health Board, Denbigh, United Kingdom

## Abstract

**Aims:**

To analyse responses from a survey sent to current resident/SAS doctors and consultants who had received the updated induction handbook and also resident/SAS doctors and consultants who had received a previous version of the same.

**Methods:**

The former induction handbook was updated after receiving feedback. A 20 question survey was sent out to all the current resident/SAS doctors and consultants working across North Wales along with the updated induction handbook.The questions included yes/no, multiple-choice and free flow text options.

**Results:**

There were 30 respondents of the survey of whom 22 had used a previous version of the induction handbook. The handbook achieved 100% satisfaction rate in the following categories:

- Roles and Responsibilities.
- Admission Guidance.
- Acute Psychiatric Emergencies.
- Discharge Processes.
- Contact Details.

A very high level of satisfaction was noted in legal frameworks (97%), training essentials (93%) and practical information (93%).

Of the respondents who had used the previous version of the handbook, 81% rated it much better and 19% rated it slightly better in clarity and structure. 81% found it much better in usefulness and relevance and 80% noted it to be much improved in design and readability. 84% of the previous respondents stated it to significantly improve overall induction experience with the rest stating it to be somewhat better.

The most valued new sections were:

- Psychiatric Emergencies and Rapid Tranquillisation.
- Legal Frameworks including Mental Health Act and Mental
- Capacity Act.
- Old Age Clerking.
- Detention in Forensic Sections.

Suggestions for improvements included:

- Information on Electronic Prescribing.
- Electronic Discharge Summaries.
- Clinical Guidance/Brief Sections on:
- Alcohol and Opioid withdrawal.
- Prescribing Opiates.
- Liaison Psychiatry assessment expectation.
- Consistent Layout and Modern Formatting.

Additional subjective feedback showed a high level of gratitude from the resident doctors.

**Conclusion:**

The results from the survey highlighted an overwhelmingly positive response to the new induction handbook from resident/SAS doctors and consultants. The handbook significantly enhanced the induction experience for new resident doctors and a high level of satisfaction was seen from clinicians who had used a former version of the handbook as well.

We will continue to make the requisite amendments based on the feedback and suggestions for the next PDSA Cycle.

